# T19. MULTIMODAL IMAGING IN FIRST EPISODE PSYCHOSIS: MAGNETOENCEPHALOGRAPHY, 7T FMRI STROOP, AND 7T MRS SPECTROSCOPY

**DOI:** 10.1093/schbul/sby016.295

**Published:** 2018-04-01

**Authors:** Timothy Gawne, Greg Overbeek, Jefferey Killen, David White, Meredith Reid, Noah Salibi, Thomas Denny, Ellis Charles, Adrienne Lahti

**Affiliations:** 1 University of Alabama at Birmingham; 2 Auburn University; 3 Louisiana Tech

## Abstract

**Background:**

Schizophrenia (SZ) is an illness whose heterogeneity has impeded understanding the underlying pathophysiology. In order to better understand this heterogeneity, here we used magnetoencephalography (MEG), 7T Magnetic Resonance Spectroscopy (MRS), and 7T fMRI during the Stroop task, on the same set of patients with first episode psychosis (SZ).

**Methods:**

22 minimally treated first episode SZ and 24 healthy controls (HC) matched for age, gender, and family socio-economic status were recruited. Neurometabolite levels were obtained from the bilateral anterior cingulate cortex using 7T proton MRS with an ultra-short echo time (5 ms) STEAM sequence, and referenced to water. MEG was performed in a 4D systems 148 channel magnetometer, and both the auditory evoked potential to 40 Hz tone clicks, and the resting state (eyes closed) were recorded. The fMRI BOLD response to the Stroop task was also recorded in a 7T scanner.

**Results:**

The magnitude of the audio-evoked MEG responses to 40 Hz tone clicks was not significantly different between SZ and HC. However, many SZ showed high levels of theta-band activity during the resting state. The ratio of theta to alpha band activity in the anterior MEG sensors significantly differentiated SZ from HC, P<0.05 by t-test. MRS levels of glutamate and total NAA (tNAA), also separated HC from SZ, P<0.05, t-test. An across-groups whole brain analysis of the Stroop fMRI BOLD response to incongruent trials relative to congruent trials was performed (p < .01, FDR-corrected). The strongest signal came from a region in the left parietal (MNI, -30, -58.5, 48), and between-group analysis of the BOLD signal from a 4mm sphere surrounding this location revealed that the activation was greater for SZ than HC by t-test, P<0.05. The MEG theta/alpha ratio, and the left parietal fMRI Stroop effect, were significantly correlated, r=0.45, P=0.005. However, the fMRI Stroop was uncorrelated with the MRS tNAA (r = -0.11, P=0.491) and also uncorrelated with the MRS glutamate levels (r = -0.16, P=0.334).

**Discussion:**

We speculate that the MEG and fMRI data, and the MRS neurometabolite levels, may reflect two relatively independent underlying pathological mechanisms in SZ. Possibly the MEG and fMRI results are indicative of dysfunction in long-range cortical-cortical networks, while the MRS data is more indicative of local neurometabolic dysfunction. Further exploration of SZ using multiple imaging modalities on the same subjects may help to untangle the underlying pathophysiological basis of the heterogeneity of this disorder.

